# Structural optimization of multistage depressurization sleeve of axial flow control valve based on Stacking integrated learning

**DOI:** 10.1038/s41598-024-58178-5

**Published:** 2024-03-29

**Authors:** Shuxun Li, Guolong Deng, Yinggang Hu, Mengyao Yu, Tingqian Ma

**Affiliations:** College of Petroleum and Chemical Engineering, Lanzhou University of Technology, Lanzhou, 730050 Gansu People’s Republic of China

**Keywords:** Axial flow control valve, Fusion model, Parameter optimization, Particle swarm optimization, Engineering, Mechanical engineering

## Abstract

Due to the requirements of the working environment, the marine axial flow control valve needs to reduce the noise as much as possible while ensuring the flow capacity to meet the requirements. To improve the noise reduction effect of the axial flow control valve, this paper proposes a Stacking integrated learning combined with particle swarm optimization (PSO) method to optimize a multi-stage step-down sleeve of the axial flow control valve. The liquid dynamic noise and flow value of the axial flow control valve are predicted by computational fluid dynamics. Based on the preliminary evaluation of its performance, the structural parameters of the multi-stage pressure-reducing sleeve are parameterized by three-dimensional modeling software. The range of design variables is constrained to form the design space, and the design space is sampled by the optimal Latin hypercube method to form the sample space. An automated solution platform is built to solve noise and flow values under different structural parameters. The Stacking method is used to fuse the three base learners of decision tree regression, Kriging, and support vector regression to obtain a structural optimization fusion model with better prediction accuracy, and the accuracy of the fusion model is evaluated by three different error metrics of coefficient of determination (*R*^2^), Root Mean Squared Error, and Mean Absolute Error. Then the PSO particle swarm optimization algorithm is used to optimize the fusion model to obtain the optimal structural parameter combination. The optimized multi-stage depressurization structure parameters are as follows: hole diameter *t*_1_ = 3.8 mm, hole spacing *t*_2_ = 1 mm, hole drawing angle *t*_3_ = 6.4°, hole depth *t*_4_ = 3.4 mm, and two-layer throttling sleeve spacing *t*_5_ = 4 mm. The results show that the peak sound pressure level of the noise before and after optimization is 91.32 dB(A) and 78.2 dB(A), respectively, which is about 14.4% lower than that before optimization. The optimized flow characteristic curve still maintains the percentage flow characteristic and meets the requirement of flow capacity *K*_*v*_ ≥ 60 at the maximum opening. The optimization method provides a reference for the structural optimization of the axial flow control valve.

## Introduction

Noise is one of the sources of environmental pollution, and its importance comes from its significant impact on all human and biological health. Excessive noise can cause a gradual decline in hearing and can also cause negative psychological effects^1,2^. There are many noise sources, including industrial activities, road traffic, etc. Among them, industrial activities are the main source of noise and one of the most important noise sources affecting human health^3^. Pipeline transportation has the advantages of safety, reliability, continuity, and a high degree of automation. It is one of the main ways of liquid transportation and an indispensable transportation infrastructure for energy systems. It is of great significance to study how to reduce the noise of pipeline systems^4,5^.

The valve is called the throat of the pipeline system. It is not only the source of vibration noise in the pipeline system but also an important source of hydrodynamic noise in the pipeline system^6^. The axial flow control valve is a new type of valve. The position of the valve core and the sleeve is changed based on retaining the advantages of traditional control valves. The sleeve and the valve core are placed in the inner cavity of the valve body along the flow channel. The flow direction of the fluid does not change. It has the characteristics of small flow resistance, large flow adjustment range, and rapid opening and closing. It is an economical and energy-saving control valve^7^. The research on high-performance similar products in China is developing rapidly. CFD high-precision simulation of its performance and research on noise reduction methods have become the key technologies for the development of the control valve industry.

With the development of computational fluid dynamics, the numerical simulation flow field prediction method for the control valve is becoming more and more mature. Domestic and foreign scholars have conducted a lot of research on the structural optimization of the control valve based on CFD numerical simulation.

Some scholars have optimized the structure of the throttling element by studying the throttling characteristics of the throttling element of the control valve. Zhang et al.^8^ used CFD to analyze the sensitivity of the throttling characteristic curve under multiphase flow conditions and optimize the geometric structure of the regulating valve spool. Zhang et al.^9^ designed a bionic structure at the throttle to suppress cavitation and improve the service life of the regulating valve, and optimized the parameters of the bionic structure. Lin^10^ studied the flow characteristics of the V-valve during the opening and closing process through experiments and numerical simulation, especially the influence of different cone angles on the valve regulation process.

The problems of fluid excitation, high-pressure drop, and cavitation caused by the high-velocity medium in the control valve have always been the pain points of industry development. The use of multi-stage pressure-reducing orifice plates can well suppress these problems. Therefore, many scholars are committed to studying the flow characteristics of multi-stage depressurization control valves. Fu-qing Chen^11^ revealed the hydrodynamic characteristics of the superheated steam flow in the multi-stage high-pressure reducing valve through experiments and numerical methods and studied the influence of typical parameters on the pressure drop and Mach number. The orthogonal design method of array L_9_ (3^4^) was used to realize the optimal design of the throttling element. Xu^12^ introduced a porous sleeve valve with a secondary pressure reduction function, studied the flow resistance, and established the relationship between the flow rate, flow area, and flow resistance coefficient of the valve. Geng^13^ used the user-defined function (UDF) to simulate the transient opening and closing process of the labyrinth regulating valve based on dynamic mesh technology. Zhang^14^ optimized the design of the regulator and analyzed and evaluated the influencing factors of irregular flow by numerical simulation to determine the final structure of the regulator. Although many scholars have designed and optimized the structure of the control valve, there is a lack of research on the comprehensive consideration of noise and flow characteristics, and the use of manual optimization design can not grasp the influence of the structure on the performance. The optimization design is based on the experience of the designer, and the efficiency is too low.

With the development of machine learning technology, using machine learning methods for engineering optimization has become a research hotspot^15,16^. Prabhakar Sharma^17^ introduced the AI-based prognostic modeling and performance optimization of the CI engine using biodiesel-diesel blends. A large number of scholars have done a lot of research on intelligent algorithms such as kriging, decision trees, and PSO particle swarm optimization in engineering optimization problems. Jiachang Qian^18^ proposed a general sequential constraint updating method based on the confidence interval of the Kriging surrogate model (SCU-CI) to solve the prediction error between the surrogate model and the actual constraint in the Kriging surrogate model aided engineering optimization design. Shuxun Li^19^ based on a surrogate optimization algorithm to optimize the profile of a V-type regulating ball valve. Marcin Czajkowski^20^ provides a detailed introduction to decision trees, discusses different representations of decision trees applied to regression problems, validates five evolutionary tree induction factors with different tree representations, and shows their advantages and disadvantages. Zheng^21^ discussed and improved the particle swarm optimization (PSO) algorithm in detail. The improved PSO algorithm was used to optimize the hull shape of the engineering ship to reduce the wave resistance coefficient under static constraints. These studies provide an analytical basis for the analysis of this paper. At present, most of the optimization methods adopt a single surrogate model or artificial optimization design. The single surrogate model has low prediction accuracy and is easy to fall into the local optimum.

To improve the noise reduction effect of the axial flow regulating valve and improve the rationality of the structural design, this paper uses a Stacking integrated learning combined with the PSO particle swarm optimization method to optimize the structure of the initial axial flow control valve. The automatic solution platform is built by Isight combined with SolidWorks modeling software and Fluent flow field simulation software to improve the efficiency of optimization design. The Stacking model fusion method is used to construct the structural optimization surrogate model, and the performance evaluation of the fusion model is completed through a variety of error metrics. Combined with the PSO particle swarm global search algorithm, the multi-stage step-down structure of the axial flow control valve is optimized, and the multi-stage step-down structure parameters with lower noise level is obtained. This method can provide a reference for the structural optimization of axial flow control valves.

## Structure and working principle

**Figure 1:**
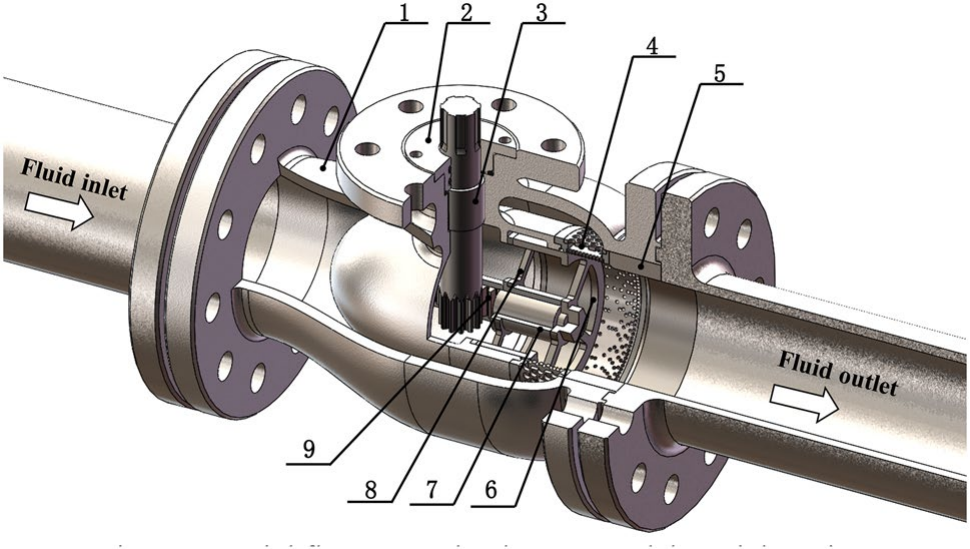
Axial flow control valve 3D model partial section.

The structure of the axial flow control valve is shown in Fig. 1, mainly including valve body 1, anti-blow-out gland 2, gear shaft 3, anechoic pressure reduction assembly 4, positioning sleeve 5, valve spool 6, guide sleeve 7, the support frame 8 and rack shaft 9. The rack and pinion drive mechanism is used to adjust the spool 6 position.

**Figure 2:**
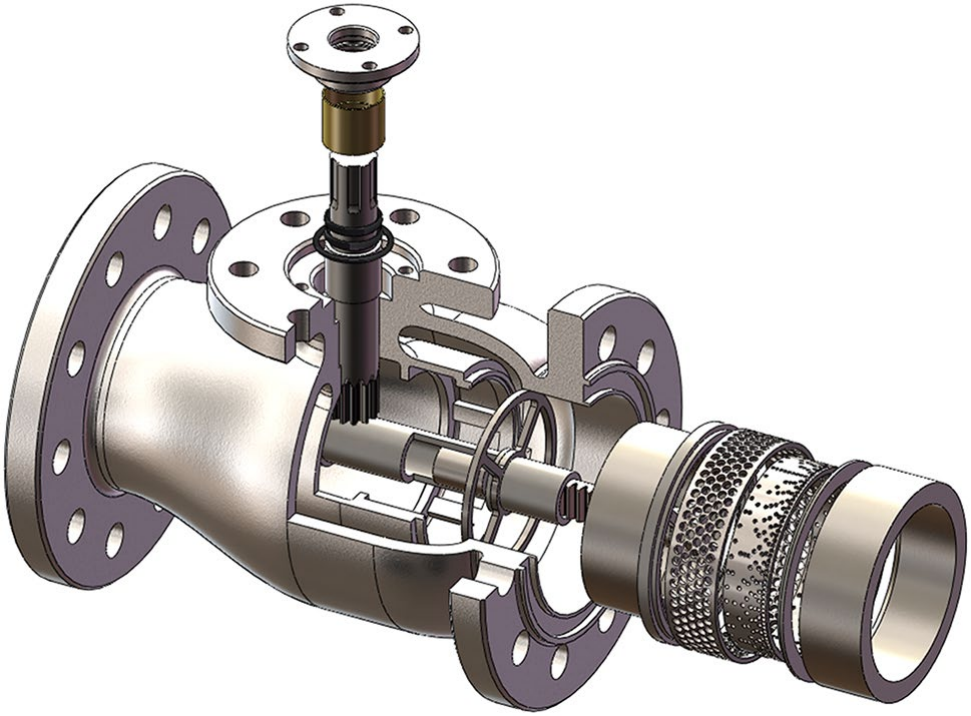
Exploded sketch of the axial flow control valve 3D model.

The axial flow control valve is composed of the valve body, sealing assembly, two-stage sleeve (including the uniform opening sleeve and percentage opening sleeve), valve core, gear shaft, rack shaft, and positioning sleeve. The exploded sketch of the axial flow control valve 3D model is shown in Fig. 2. The drive unit is connected to the spline end of the gear shaft, and the spool position is adjusted by driving the gear shaft to drive the rack, to reduce the flow area, and achieve control of fluid flow, and low noise at full opening.

The axial flow control valve multi-stage pressure reduction structure is initially designed for 2 stages, if its pressure reduction capacity or noise reduction capacity is not enough, the intelligent optimization algorithm can be used to further optimize the number of stages or structure of multi-stage pressure reduction structure sleeve.

## Simulation method

To evaluate the performance of the initial axial flow control valve and determine the optimization goal, the internal flow field information and liquid dynamic noise are simulated and analyzed by CFD.

The fluid flow follows the Navier–Stokes equation. The governing equations for the application of compressible viscous fluid flow are as follows:

① Energy-conservation equation

1 $$\frac{\partial (\rho T)}{{\partial t}} + {\text{div(}}\rho uT{\text{) = div}}\left( {\frac{k}{{C_{p} }}{\text{grad}}\;T} \right) + S_{T}$$

Among them, *k* is the heat transfer coefficient, W/m·K; *T* is the temperature, ℃; *C*_*p*_ is the specific heat capacity, kJ/(kg·K); *S*_*T*_ is a viscous dissipation term; *ρ* is the medium density, kg/m^3^; *u* is the velocity of medium flow, m/s.

② Momentum-conservation equation

2 $$\frac{\partial }{\partial t}\left( {\rho u_{i} } \right) + \frac{\partial }{{\partial x_{j} }}\left( {\rho u_{i} u_{j} } \right) = - \frac{\partial p}{{\partial x_{i} }} + \frac{{\partial \tau_{ij} }}{{\partial x_{i} }} + \rho g_{i} + F_{i}$$

Among them, *p* is the static pressure on the micro-element, Pa; *τ*_*ij*_ is the stress tensor; *g*_*i*_ and *F*_*i*_ are the gravity and external volume force in the* i* direction, N.

③ mass-conservation equation

3 $$\frac{\partial \rho }{{\partial t}} + \frac{\partial }{{\partial x_{i} }}\left( {\rho u_{i} } \right) = 0$$

Among them, *ρ* is fluid density, kg/m^3^.

### CFD numerical simulation

The Large eddy simulation (LES) method is used to simulate and analyze the internal flow field of a preliminary axial flow control valve.

#### Establishment of flow channel model of the axial flow control valve

**Figure 3:**
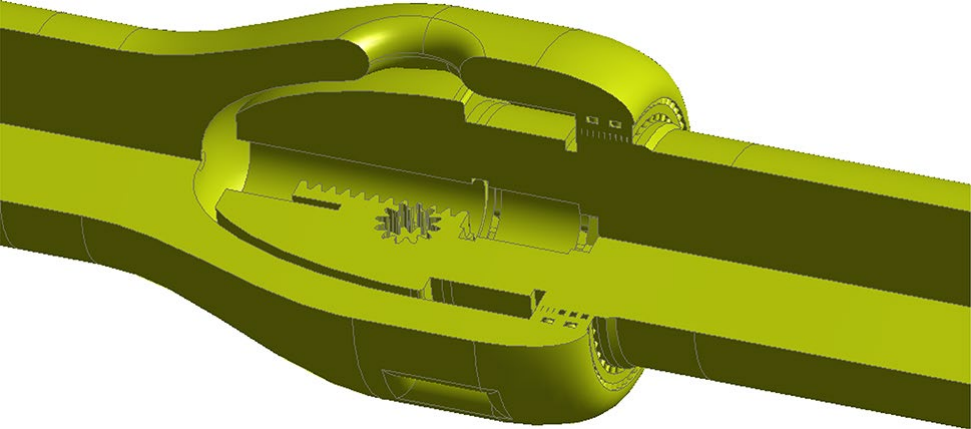
Flow channel model of the axial flow control valve.

Using three-dimensional modeling software to establish solid models with different openings. To ensure full development of turbulence in the valve, add five times the nominal diameter length of straight piping before the valve and ten times after the valve. To ensure the convergence of the calculation and improve the efficiency of the calculation, on the premise of ensuring the accuracy of the calculation, the process chamfering, chamfering, and structures that have little impact on the flow analysis in the 3D model are simplified. Based on the simplified 3D solid model, the flow channel model is generated by reverse modeling. As shown in Fig. 3.

#### Grid delineation and irrelevance verification

**Figure 4:**
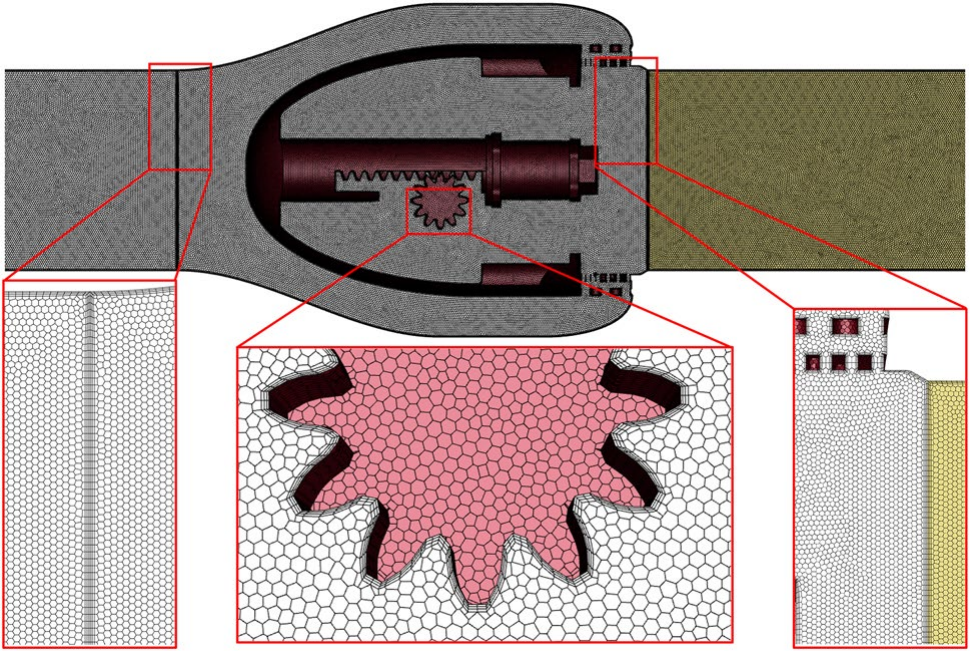
Axial flow control valve overall mesh division and local details.

The axial flow control valve flow channel model mesh is generated by Fluent Meshing software. Fluent Meshing software uses mosaic meshing techniques using conformal polyhedral elements to enable it to quickly delineate unstructured meshes of complex structural fluids. The axial flow control valve flow path is divided into three areas, which are the straight pipe area before the valve, the main basin, and the straight pipe area after the valve. The overall flow channel grid and sub-regional grid division are shown in Fig. 4.

As shown in Fig. 4, two adjacent mesh regions are connected using a split interface form, and the stitching between polyhedral meshes is processed with conformal mapping, and their nodes correspond to each other. By imposing size constraints on the smallest geometric features, the overall mesh is smoothed over from small to large basins, facilitating the division of the boundary layer mesh over the entire fluid domain and capturing the flow information at the solid walls.

The grid quality of each region is evaluated by the maximum skewness, and the evaluation results are shown in Table 1. The requirement of maximum skewness less than 0.95 when performing CFD simulation is satisfied.

**Table 1:** Axial flow control valve grid quality assessment.

	Straight pipe area before the valve	Main watershed	Straight after the valve domain
Maximum skew rate	0.339	0.926	0.316

**Figure 5:**
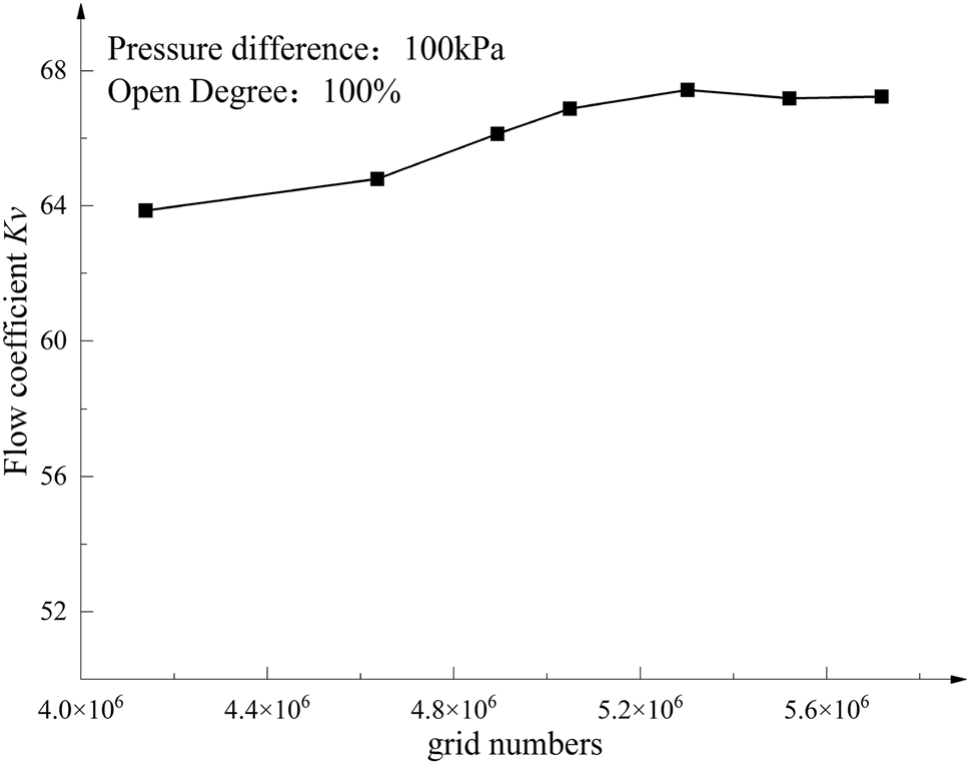
Grid-independence verification.

The grid-independence test with the flow rate value of the axial flow control valve at full opening as the target is shown in Fig. 5. From the grid-independent verification results in Fig. 5, it can be seen that when the grid number is greater than 4.8 million, the flow value tends to stabilize as the grid number increases by 16%, while the flow coefficient value *K*_*v*_ increases by 2.3%. Considering the accuracy, calculation efficiency, and other factors, the grid of about 4.6 million is selected for CFD simulation calculation.

#### Determination of numerical simulation boundary conditions and calculation of solution for axial flow control valve

Numerical simulation based on the general-purpose solver Fluent, the solver type is set to pressure type, and the solution process is based on the SIMPLE algorithm. The LES method is used for simulation, and the boundary conditions for simulation calculation are selected as pressure inlet and pressure outlet, 4 MPa before the valve and 0.1 MPa after the valve. To ensure simulation accuracy, different surface roughness is set on different wall surfaces. To accelerate the convergence, the "Least Square Cell Based" format is chosen for the gradient term, the "Second Order Windward" format is chosen for the pressure term, and the "First Order Windward" format is chosen for the momentum, turbulent kinetic energy, and turbulent dissipation rate terms. Meanwhile, to maintain the stability of the calculation, the default value of the relaxation factor is kept for each calculated parameter.

The convergence criterion in the solution process is: ①Each residual curve is below 1 × 10^–6^; ②The change rate of valve outlet flow and valve outlet center velocity is less than 1 ‰. It should be noted that only the mesh type differs in the different discretization strategies, while the mesh size (including body mesh size and surface mesh size) is the same. It should also be noted that the most severe throttling process inside the axial flow control valve occurs near the multi-stage step-down component. Therefore, to capture the flow details, additional mesh refinement is required on the wall and its adjacent surfaces of the multi-stage depressurization component. Normal-temperature water is selected as the simulation medium, and its physical parameters are shown in Table 2.

**Table 2:** Physical parameters of room temperature water.

Pressure (atm)	Temperature ℃	Density (kg/m^3^)	Isobaric-specific heat capacity (kJ/(kg K))	Dynamic viscosity (Pa s)
1	25	997	4.1819	8.9008e−04

#### Flow field analysis of the initial axial flow control valve

**Figure 6:**
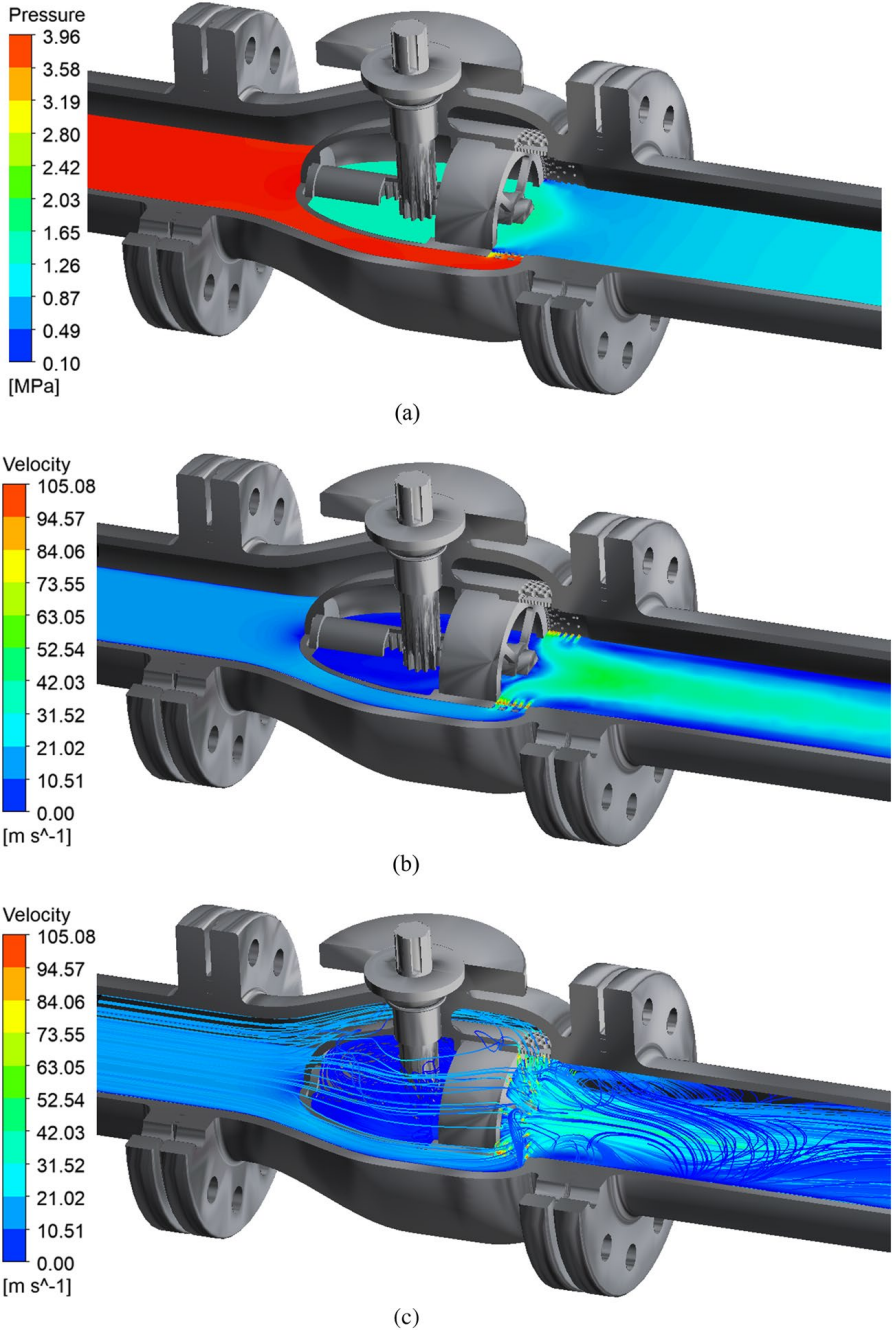
Simulation results at 100% opening. (**a**) The actual working condition 100 % opening pressure cloud diagram. (**b**) The actual working condition 100 % opening speed cloud diagram. (**c**) The actual working condition 100 % opening speed streamline diagram.

Taking full opening as an example, the internal flow field of the axial flow control valve is analyzed. The internal flow field of the initial axial flow control valve under full opening is shown in Fig. 6:

The simulation results of the actual working conditions at 100% opening of the axial flow control valve obtained from Fig. 6 show that: From the pressure distribution cloud diagram of Fig. 6a, it can be seen that the fluid pressure decreases rapidly from 4 MPa to about 0.6 MPa after the fluid passes through the multi-stage decompression sleeve of the valve. From the velocity streamline distribution cloud diagram in Fig. 6c, it can be seen that the fluid converges at the middle cavity behind the valve, and the peak velocity of the fluid in the multi-stage pressure-reducing sleeve reaches 105.08 m/s. After the fluid flows out of the pressure-reducing sleeve, the fluid streamline begins to stagger, indicating that the fluid intersects and collides, which leads to a rapid decrease in the pressure and velocity of the fluid medium, and then flows to the back of the valve.

### CFD noise prediction

The Ffowcs Williams and Hawkings (FW-H) method is used for acoustic calculation based on unsteady CFD results to evaluate the acoustic performance of the initial axial flow control valve.

#### Mechanism of liquid noise generation

**Figure 7:**
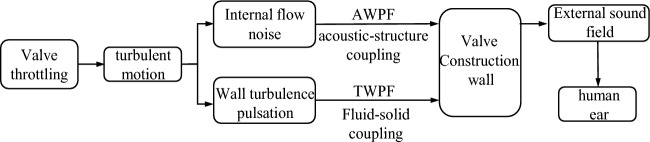
Noise generation mechanism of axial flow control valve.

The noise generation mechanism of the axial flow control valve is shown in Fig. 7. When the axial flow control valve is working, the fluid medium is in a turbulent state. Due to the throttling effect of the multi-stage pressure-reducing sleeve of the axial flow control valve, a small amount of vortex will be continuously formed and shed, resulting in pressure pulsation, which will propagate around through the fluid medium. The pressure pulsation in the turbulent medium contains an audible acoustic frequency part, which radiates the flow noise and the sound-solid coupling of the inner wall surface of the valve and valve-controlled pipe system to generate vibration noise. In addition to the noise generated by internal turbulence, the fluid medium also includes the vibration radiation noise generated by the fluid–solid coupling between the fluid medium and the inner wall of the valve and valve-controlled pipe system.

#### Calculation method of liquid noise

The noise of the axial flow control valve is induced by the flow of the internal medium of the axial flow control valve. The FW-H method is used to perform acoustic calculations based on unsteady CFD results^22,23^.

Firstly, the sound source term is calculated according to the LES. The Fluent software can be used for the unsteady calculation to obtain the flow field information of the liquid dynamic noise sound source integral surface of the internal flow of the axial flow control valve. Then, the FW-H noise analysis module provided by Fluent software is used to transform the flow field information of the liquid dynamic noise sound source integral surface into the noise source through the generalized FW-H equation, and then the wave equation is solved to obtain the noise at the far field receiving point.

**Figure 8:**
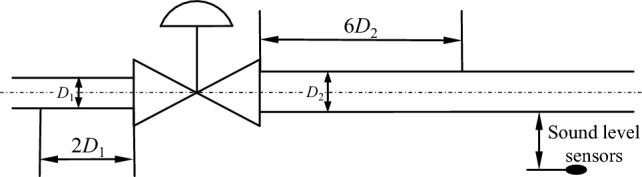
Schematic diagram of the location of the noise reception point.

Fluent software is used to simulate the typical opening of the axial flow control valve (10%, 20%, 30%, …, 90%, 100%). The time step is 1 × 10–4 s when simulating the transient flow field. Select the Acoustic acoustic solution model and activate the FW-H noise analysis module. The monitoring point is set at 1 m behind the valve and 1 m away from the outer wall of the pipeline. The location of the noise monitoring point is shown in Fig. 8.

#### Analysis of the noise prediction results

**Figure 9:**
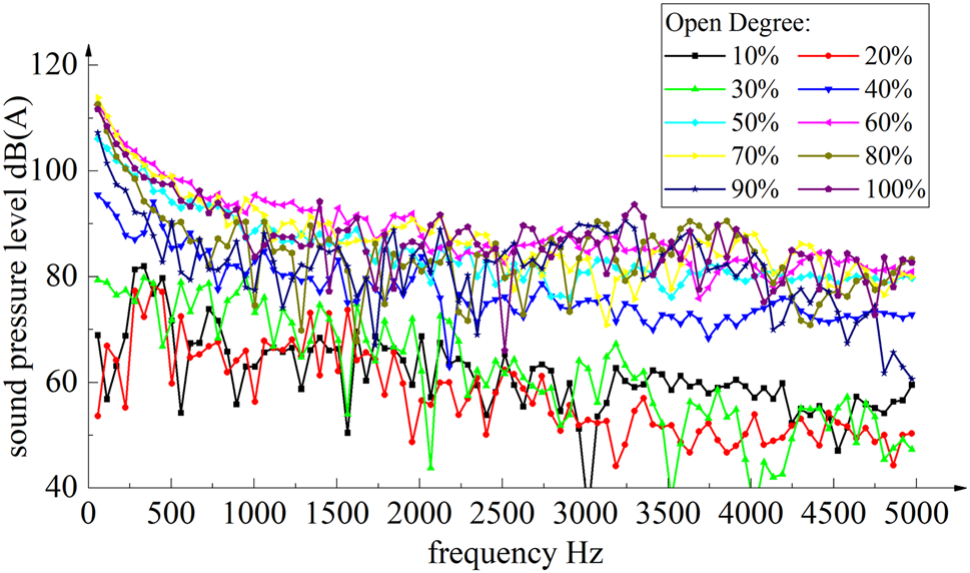
Sound pressure level and frequency curve under a typical opening.

The sampling frequency of the noise is 20–5000 Hz. The sound pressure levels of the noise monitoring points at different openings of the axial flow control valve are calculated respectively. The relationship between the sound pressure level and the frequency of the receiving point is obtained by Fourier transform^24^. The sound pressure level and frequency curve under some typical openings are shown in Fig. 9.

After obtaining the relationship between the sound pressure level and the frequency of the receiving point by Fourier transform, the noise value *L*_pAe,1m_ at 1 m outside the valve is calculated.

Firstly, the pressure difference ratio *x*_*F*_ is obtained from the pressure difference *p*_1_–*p*_2_ and the saturated vapor pressure *p*_*v*_:

4 $$x_{F} = \frac{{p_{1} - p_{2} }}{{p_{1} - p_{v} }}$$

Then the modified pressure drop ratio *x*_*Fzp*1_ is obtained from the pressure drop ratio *x*_*F*_.

5 $$x_{Fzp1} = x_{F} \left( {\frac{{6 \times 10^{5} }}{{p_{1} }}} \right)^{0.125}$$

Then compare the pressure difference ratio xf and the modified pressure difference ratio to determine whether the noise is turbulent noise or the internal sound pressure level *L*_*pi*_ of cavitation noise:

6 $$L_{pi} = 10\lg \left( {\frac{{3.2 \times 10^{9} W_{a} \rho_{L} c_{L} }}{{D_{i}^{2} }}} \right)$$

The noise level at 1 m outside the turbulence valve *L*_*pAe*,1*m*_ is:

7 $$L_{pAe,1m} = L_{pi} + TL_{{{\text{turb}}/{\text{cav}}}} - 10\lg \left( {\frac{{D_{i} + 2t_{p} + 2}}{{D_{i} + 2t_{p} }}} \right)$$

Finally, the weighted noise *L*_*pAe*_ calculation formula at the receiving point can be obtained:

8 $$L_{pAe} = 10\lg \left( {10^{{\frac{{L_{pAe1} }}{10}}} + 10^{{^{{\frac{{L_{pAe2} }}{10}}} }} + \cdots } \right)$$

**Figure 10:**
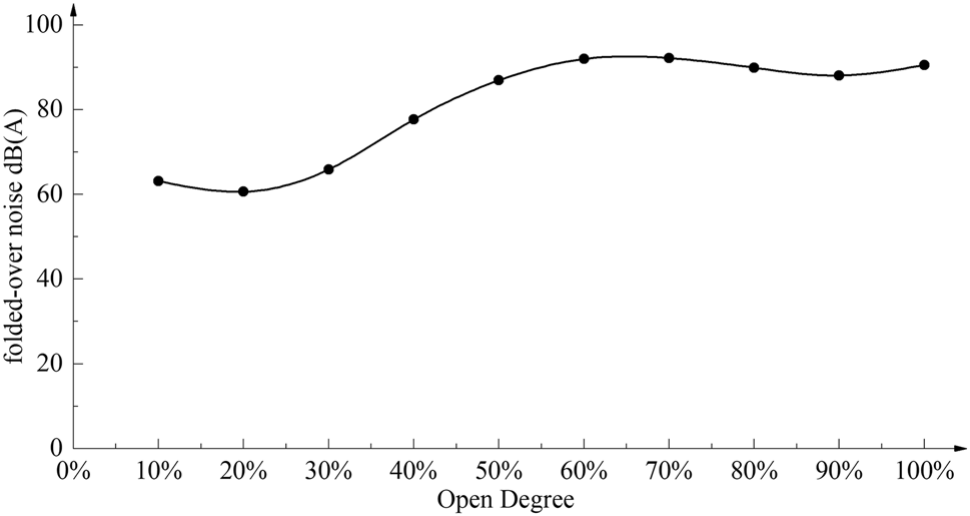
Superimposed weighted noise at monitoring points at different openings.

The superimposed weighted noise at the receiving point of the axial flow control valve at different openings is calculated according to Eq. (8), and the results of the superimposed weighted noise results at different openings are shown in Fig. 10.

As shown in Fig. 10, with the increase of opening degree, the superposition noise of the axial flow control valve shows an increasing trend, reaching a maximum value of about 91.32 dB(A) at about 65% opening degree. The noise value is at a high level. Due to the special use environment of the axial flow control valve, the peak noise sound pressure level at the full opening is required to be as low as possible. Therefore, it is necessary to optimize the outer noise reduction sleeve of the multistage step-down structure of the axial flow control valve.

## Optimization for design

Aiming at the problem of high noise level and large noise peak of the initial axial flow control valve, a Stacking ensemble learning combined with PSO particle swarm optimization method for the optimization design of the multi-stage pressure-reducing sleeve of the axial flow control valve is proposed.

### Optimization goals and processes

**Figure 11:**
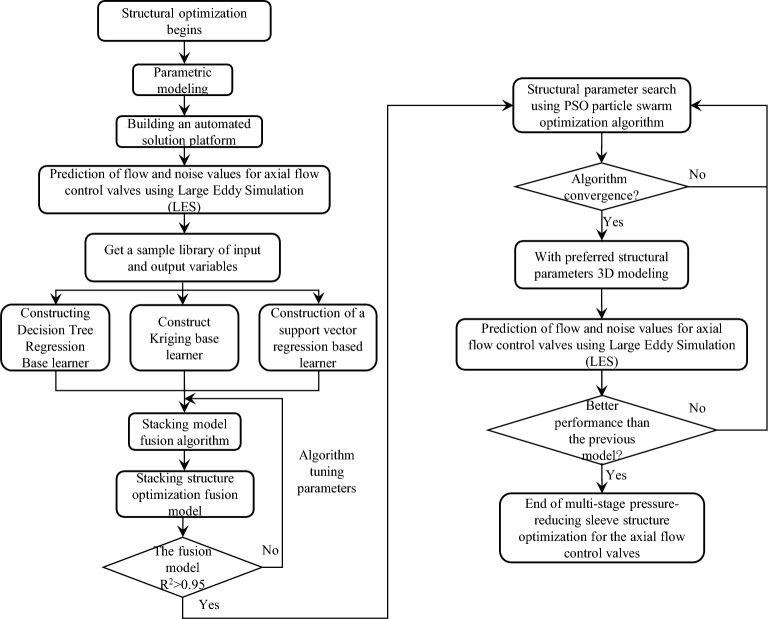
Flow chart of multi-stage depressurization structure optimization of the axial flow control valve.

The design goal for axial flow control valves is low noise at full opening. The axial flow control valve has the largest working pressure difference at a small opening, the flow area is small, the throttling is the most serious, and the adverse situation of high noise is prone to occur. Firstly, the noise superposition sound pressure level under the typical opening of the axial flow control valve is simulated to further determine whether the noise value under the full opening is less than 75 dB. If it is not satisfied, it is necessary to optimize the multi-stage step-down structure, and at the same time, the use requirement of flow capacity *K*_*v*_ ≥ 60 at the maximum opening should be guaranteed. Therefore, the structural optimization of the axial flow control valve is a typical multi-objective optimization problem. The optimization process of the multi-stage step-down structure of the axial flow control valve is shown in Fig. 11.

### Parametric modeling

Before constructing the surrogate model of axial flow control valve structure optimization, it is necessary to simulate the multi-stage pressure-reducing sleeves of different structures many times. Firstly, the 3D solid model of the axial flow control valve should be parametrically modeled, and then the automatic solution platform should be constructed for the automatic solution. Parametric modeling refers to the application of a series of parameters to define some characteristics of the product when the designer is designing the product. As long as the parameter value is changed, the structural characteristics of the product can be changed, and the intelligent optimization algorithm is applied to improve its structure.

**Figure 12:**
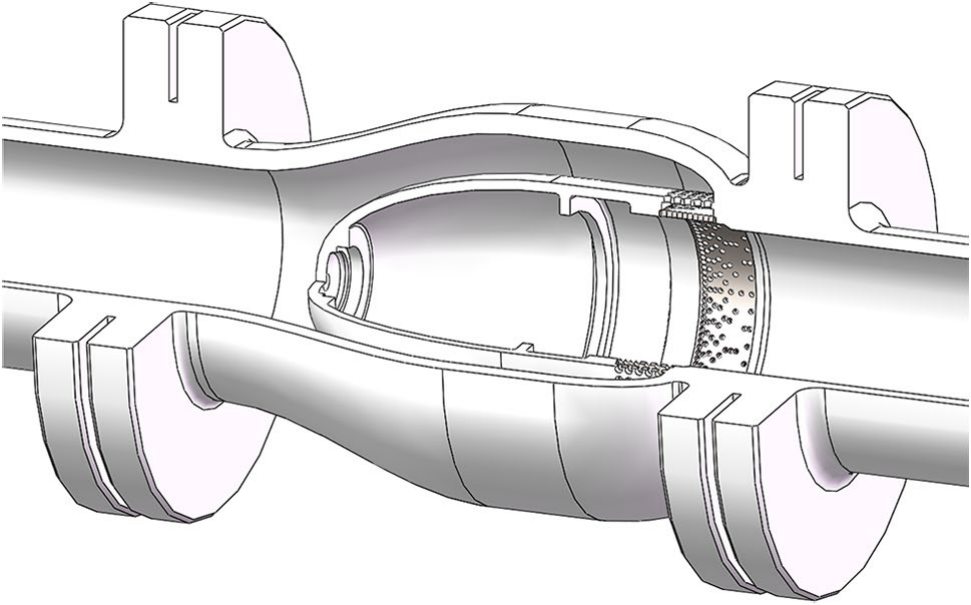
Simplified solid model for noise calculation of axial flow control valve.

To reduce the calculation amount of a single calculation, the axial flow control valve model is simplified, and the valve core, transmission components, and other components that do not affect the overall flow are suppressed. The simplified model is shown in Fig. 12, and SolidWorks is used to parametrically model the axial flow control valve.

**Figure 13:**
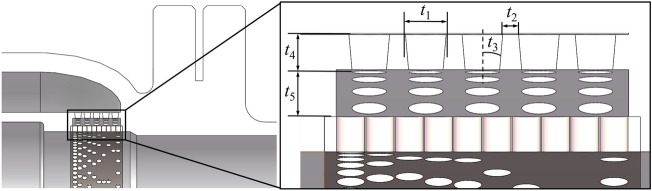
Parametric modeling of multi-stage step-down components.

To explore the influence of the five variables of the outer sleeve's throttling hole diameter *t*_1_, hole spacing *t*_2_, hole drafting angle *t*_3_, hole depth *t*_4_, and two-layer throttling sleeve spacing *t*_5_ on the flow performance of the axial flow control valve and the noise reduction ability of the outer noise reduction sleeve, the parametric modeling of the outer noise reduction sleeve of the axial flow control valve is shown in Fig. 13.

**Figure 14:**
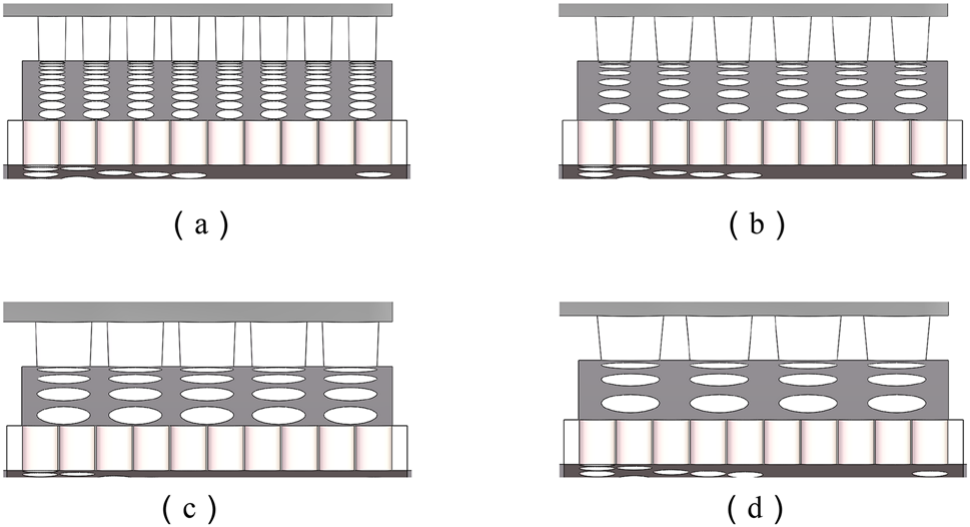
Reconstruction model of typical structural parameters of multistage step-down sleeve. (**a**) *t*_1_=2mm,*t*_3_=1°. (**b**) *t*_1_=3mm,*t*_3_=4°. (**c**) *t*_1_=4mm,*t*_3_=2°. (**d**) *t*_1_=5mm,*t*_3_=5°.

The spool stroke of the axial flow control valve is 25 mm. When the diameter of the outer sleeve orifice is changed, the equation control model in SolidWorks is used to automatically reconstruct the model. The automatic reconstruction model of typical orifice diameter *t*_1_ and hole drawing angle *t*_3_ is shown in Fig. 14.

The five variables of the Throttle hole diameter *t*_1_, the hole spacing *t*_2_, the hole extraction angle *t*_3_, the hole depth *t*_4_, and the Throttle sleeve spacing *t*_5_ of the outer sleeve are used as the design variables to construct the axial flow control valve structure optimization surrogate model.

The prediction accuracy of the proxy model depends on whether the design variables used to construct the proxy model can truly reflect the model's performance. To ensure the correctness and authenticity of the structural optimization sample database, the ranges of the design variables were constrained, and the constrained ranges of the five design variables are shown in Table 3. The optimal Latin hypercube design was used to sample the design space to ensure uniformity of sample space points, improve prediction accuracy, and reduce the prediction blind area. 110 sample points were sampled for the design, and some of the optimal Latin hypercube sampling results are shown in Table 4.

**Table 3:** Range of design variable constraints.

Design variables	Initial value	Lower limit	Upper limit
Throttle hole diameter *t*_1_ (mm)	4	2	6
Hole spacing *t*_2_ (mm)	0.5	0.1	1
hole extraction angle *t*_3_ (°)	0	0	5
hole depth *t*_4_ (mm)	3	3	5
Throttle sleeve spacing *t*_5_ (mm)	4	3	5

**Table 4:** Optimal Latin hypercube sampling results of structural optimization database.

Throttle hole diameter *t*_1_ (mm)	Hole spacing *t*_2_ (mm)	hole extraction angle *t*_3_ (°)	hole depth *t*_4_ (mm)	Throttle sleeve spacing *t*_5_ (mm)
3.62	0.1225	1.9865	3.23	4.07
3.42	0.1585	0.5655	3.15	4.55
4.14	0.8965	4.7305	3.37	3.87
2.74	0.1315	3.7995	4.37	4.03
5.86	0.2215	3.3095	4.69	4.25
3.5	0.7165	3.3585	4.27	3.13
4.86	0.8875	1.8395	3.09	4.27
4.3	0.8785	1.0065	3.53	3.53
2.78	0.7795	4.4365	3.73	4.95
5.5	0.4015	0.9085	4.05	3.11
5.78	0.9775	0.1245	3.75	3.77

### Automatic solving platform for sample axial flow control valve structure optimization library.

Isight software has rich multi-software interfaces and high-degree-of-freedom integration methods. It can organize all design processes into a unified logical framework to realize the self-starting of modeling software numerical simulation software and interface data interaction. The output value of the sample data is automatically calculated, so that the entire process of obtaining the target value is automated and the iteration cycle is shortened. Therefore, the Isight software integration platform is used to couple SolidWorks with Fluent software of ANSYS Workbench to realize the automatic solution of the software to construct the sample database of axial flow control valve structure optimization. Then the calculated sample database is exported as a .csv file, and the five design parameters are expressed as column vector *T* = [*t*_1_ *t*_2_ *t*_3_ *t*_4_ *t*_5_] ^*T*^ as the input of the Python program to construct the surrogate model. The Jupyter Notebook software was used to complete the construction and optimization of the prediction model.

### Multi-learner model fusion methods

The main idea of the stacking algorithm is to use the primary learner to train k-base models and secondary training sets on the training set, integrate them into a strong learner, and then stack the learners on the strong learner to improve the prediction accuracy of the constructed surrogate model^25^. The Stacking algorithm is implemented as follows:

Firstly, the base prediction model (*M*_*basei*_) is trained by three machine learning algorithms: decision tree regression, Kriging surrogate model, and support vector regression.

9 $$M_{{{\text{base}}}} = \left\{ {M_{1} ,M_{2} ,M_{3} } \right\}$$

For each learner $M_{basei} ,i \in \left\{ {1,2,3} \right\}$, the *i*-th base prediction model is used to obtain the predicted value *y*_*i*_ on the *i*-th training set. Four groups of predictive values were combined to obtain the secondary training set *T*_*s*_.

10 $$\begin{aligned} T_{s} & = \left\{ {y_{1} ,y_{2} ,y_{3} } \right\} \\ y_{i} & = M_{i} (x),i = 1,2,3 \\ \end{aligned}$$

Then, based on the secondary training set *T*_*s*_, the learner is trained to obtain an integrated prediction model.

**Figure 15:**
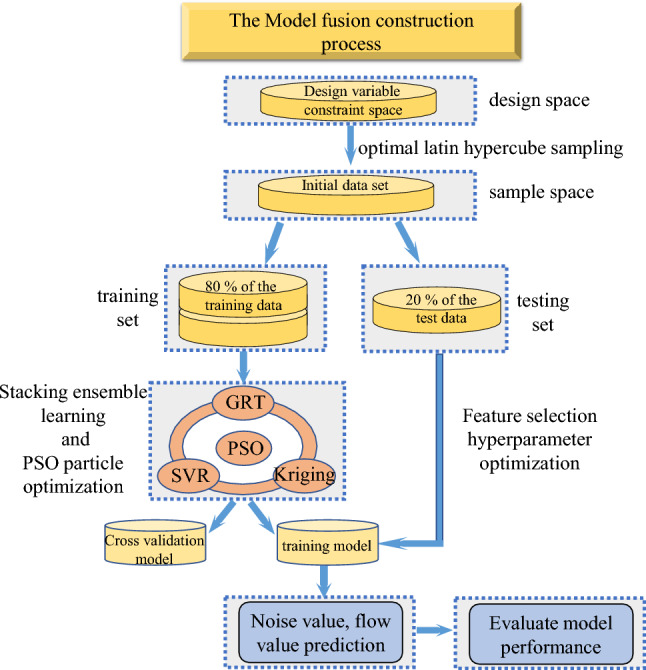
Stacking structure optimization fusion model construction process.

The whole process of implementing the agent model construction for structural optimization of the axial flow control valve using the Stacking integrated learning method is shown in Fig. 15.

The Stacking model fusion method makes full use of different learners ' different observations of data from different data space angles and data structure angles to complement each other and does not weaken the prediction accuracy of a single weak learner in theory.

### Stacking multi-model fusion process and prediction performance evaluation

The Stacking multi-model fusion method is used to integrate the three basic learners of decision tree regression, Kriging, and support vector regression to construct the surrogate model of the axial flow control valve structure optimization. Different metrics are used to evaluate the performance of the fusion model.

#### Stacking fusion model construction process

The Stacking multi-model fusion method is used to integrate the three basic learners of decision tree regression, Kriging, and support vector regression to construct the surrogate model of axial flow control valve structure optimization.

Decision tree regression provides a binomial tree for regression prediction. For the construction of the optimal regression decision tree, the squared error minimization criterion is used for feature selection, which is used to solve for the optimal output value on each part. The heuristic method is used to divide the database input space into nodes. *x* is the input variable, and the jth variable *x*^(*j*)^ and its value s are selected as the cut variable and the cut point, and two regions (*R*_1_, *R*_2_) can be defined.

11 $$\begin{aligned} R_{1} \left( {j,s} \right) & = \left\{ {\left. x \right|\;x^{(j)} \le s} \right\} \\ R_{2} \left( {j,s} \right) & = \left\{ {\left. x \right|\;x^{(j)} > s} \right\} \\ \end{aligned}$$

Kriging is used for the construction of a regression predictive agent model for the structural optimization of axial flow control valves database, which provides predicted values of the simulated response at unexperimented points and also gives a measure of prediction uncertainty^26^. The Kerriging agent model explores the unknown region of the sample space by Gaussian estimation of the unknown points and mean squared error (MSE) estimation.

Taking 100 sample points as the initial training samples, the other 10 Kriging models are used to test the trained Kriging nonlinear relationship between the optimization design variables and the optimization objectives is established by taking the noise value and flow value of the axial flow control valve as the response values.

The surrogate model of the axial flow control valve structure optimization established by the Kriging method has a predicted uncertainty of 0 at the trained point.

**Figure 16:**
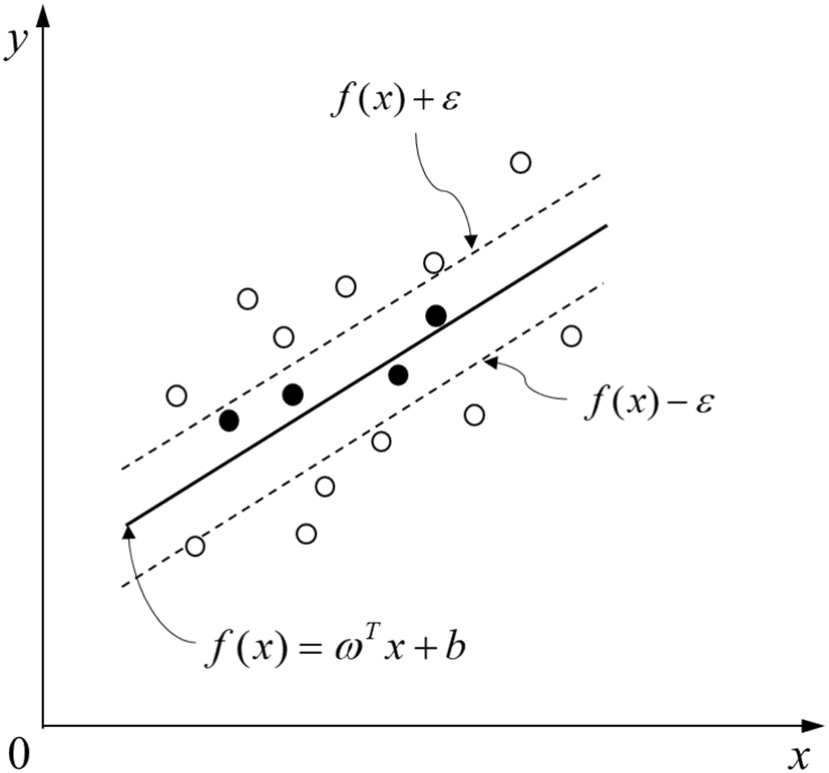
Support vector regression diagram with an interval of 2*ε.*

Support vector regression provides a loss function calculation method. For the sample (*x*, *y*), it is assumed that the deviation between *f*(*x*) and y can be tolerated at most *ε*, that is, the loss is calculated only when the absolute value of the difference between the model output *f*(*x*) and *y* is greater than *ε*^27^. As shown in Fig. 16, only training samples that fall into an interval band of width 2*ε* are considered to be correctly predicted.

**Figure 17:**
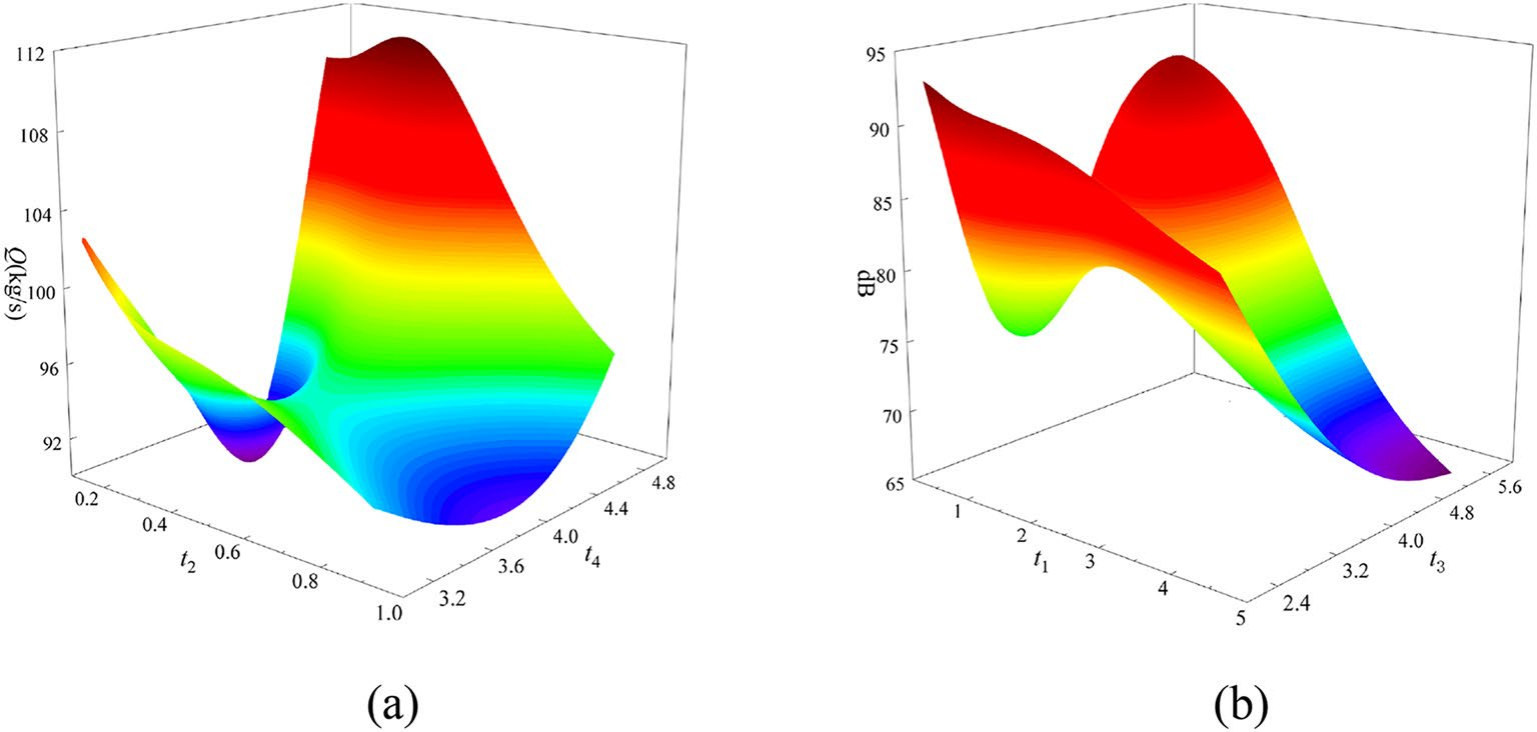
Stacking fusion surrogate model response surface. (**a**) The response surface of Q concerning *t*_2_ and *t*_4_. (**b**) The response surface of noise value dB concerning *t*_1_ and *t*_3_.

The cross-validation method is used to optimize the optimal hyperparameters of each model, and the weight coefficient of each base learner is calculated according to the inverse proportional averaging method, to ensure that the base learner with high prediction accuracy has a larger weight coefficient. The response surface of the final structural optimization approximation function is shown in Fig. 17.

#### Stacking fusion model prediction performance evaluation

For the prediction results of the Stacking fusion model, three different error metrics, *R*^2^, RMSE, and MAE, are used to evaluate the accuracy of the fusion model. The evaluation results are shown in Table 5.

**Table 5:** Stacking fusion model prediction performance evaluation metrics.

Evaluation metrics	*R*^2^	RMSE	MAE
Flow rate value	0.9734	0.2148	1.2136
Noise value	0.9876	0.2005	1.0645

As shown in Table 5, R^2^ is close to 1, and RMSE and MAE are close to 0, indicating that the prediction performance of the fusion model is good. Taking *R*^2^ as an example, the evaluation results of the mathematical model of decision tree regression structure optimization of the axial flow control valve are analyzed.

**Figure 18:**
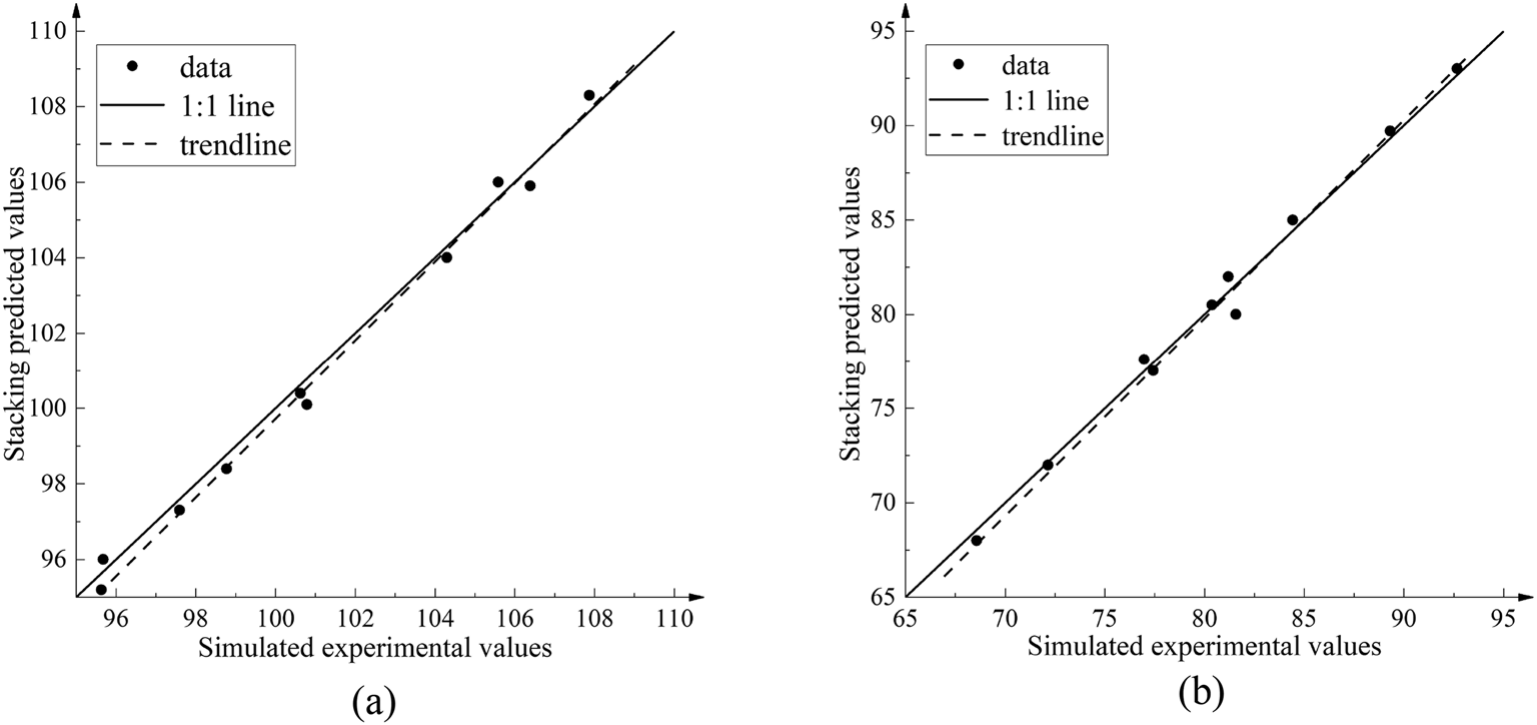
Stacking fusion agent model *R*^2^ model evaluation results. (**a**) Flow rate value *R*^2^curve. (**b**) Noise value *R*^2^ curve.

The method of calculating *R*^2^ is used to evaluate the mathematical model of decision tree regression structure optimization of the axial flow control valve, and the results are shown in Fig. 18.

The model evaluation result of the Stacking fusion surrogate model is that the traffic value *R*^2^ is 0.9734 and the noise value *R*^2^ is 0.9876. From the *R*^2^ curve of the flow value in Fig. 18a, it can be seen that the trend line of the verification point intersects with the 1:1 line, but the trend is roughly the same. The 10 verification points are evenly distributed on both sides of the 1:1 line and the position is close to the 1:1 line, indicating that the Stacking fusion model has a high accuracy in predicting the flow value of the axial flow control valve. From the *R*^2^ curve of the noise value in Fig. 18b, it can be seen that the trend line of the verification point is crossed with the 1:1 line, and the trend is roughly the same and distributed near the 1:1 line. The 10 verification points are evenly distributed on both sides of the 1:1 line, indicating that the Stacking fusion model has high accuracy in predicting the noise value of the axial flow control valve.

The *R*^2^ values of the 3 base learners with the Stacking fusion agent model are shown in Table 6.

**Table 6:** Comparison of *R*^2^ determination coefficient between single learner and Stacking fusion model.

Response value	*R*^2^			
	decision tree regression	Kriging model	Support vector regression	Stacking fusion model
Flow rate value	0.8709	0.9102	0.9255	0.9734
Noise value	0.9635	0.9723	0.9848	0.9876

From Table 6, it can be seen that the prediction accuracy of the Stacking fusion surrogate model for the flow value and noise value of the axial flow control valve is slightly higher than that of the single learner using decision tree regression or Kriging or support vector regression. Therefore, the Stacking fusion agent model is used as the axial flow control valve structure optimization agent model for parameter optimization.

### Stacking fusion surrogate model optimization based on particle swarm PSO algorithm

The particle swarm optimization algorithm is a group-based random search technology. In the search process, the previous best position and the global best position are remembered. The whole search update process is the process of following the current optimal solution^28^. Each particle has a position vector *X*_*i*_ and a velocity vector *V*_*i*_ to calculate the fitness value determined by the optimization objective function. After calculating the new value of the objective function, the position and velocity of the particles will change. The particle update iteration process is shown in Eq. (12).

12 $$\begin{aligned} V_{i}^{(t + 1)} & = wV_{i}^{(t)} + c_{1} r_{1} (Pbest_{i}^{(t)} - X_{i}^{(t)} ) + c_{2} r_{2} (Gbest^{(t)} - X_{i}^{(t)} ) \\ X_{i}^{(t + 1)} & = X_{i}^{(t)} + V_{i}^{(t + 1)} \\ \end{aligned}$$

In the formula: *w* is the inertia weight scale factor, which is used to control the influence of the old speed on the new speed; *Pbest*_*i*_ is the best position to calculate the best fitness of the *i*th particle; *Gbest* is the global best particle currently sought; *c*_1_ and *c*_2_ are two constants called self-factors and global factors, which determine the weights of *Pbest*_*i*_ and *Gbest* respectively; *r*_1_ and *r*_2_ are two random numbers generated in the range of [0, 1].

**Figure 19:**
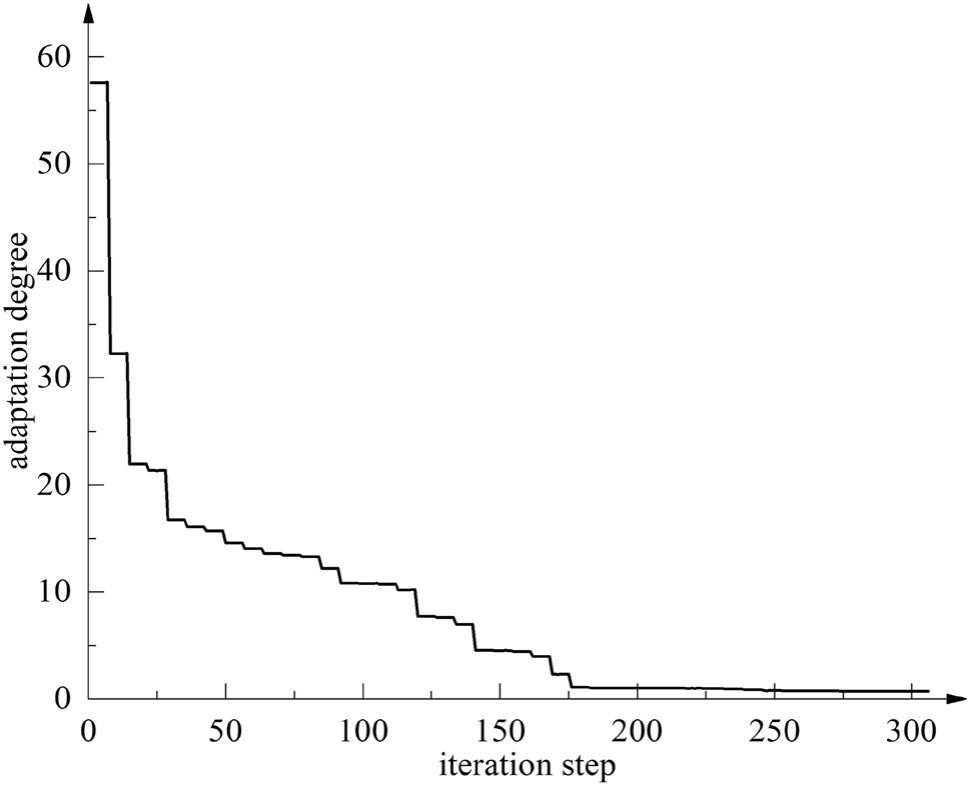
The best fitness convergence curve of the Stacking fusion model optimization process.

The PSO algorithm is a relatively mature global optimization algorithm, which has good applicability in engineering optimization problems. The particle swarm PSO global optimization algorithm is used to optimize the constructed Stacking structure optimization fusion agent model of the axial flow control valve. The particle swarm PSO optimal fitness convergence curve in the optimization process is shown in Fig. 19.

The parameters obtained by PSO global optimization are shown in Table 7. Considering the processing cost, the optimized structural parameters are rounded.

**Table 7:** Comparison of parameters before and after structural optimization.

Design variables	Before optimization	After optimization	Optimized parameters (rounding)
Throttle hole diameter (mm) *t*_1_	4	3.84	3.8
Hole spacing(mm)* t*_2_	0.5	0.98	1
Hole extraction angle (°) *t*_3_	0	6.43	6.4
Depth of hole (mm) *t*_4_	3	3.38	3.4
Throttle sleeve spacing (mm) *t*_5_	4	3.86	4

The optimized structural parameters after reasonable rounding are used for 3D modeling, and the internal flow channel and noise value under actual working conditions are simulated.

## Analysis of results

To verify the optimization results of the structural optimization method of the axial flow control valve, the structural parameters obtained by the optimization algorithm are used for three-dimensional modeling. The reverse modeling, meshing, grid independence test, and simulation methods are the same. When analyzing the internal flow field of the axial flow control valve under 100% opening, the medium is normal temperature water, and the boundary conditions are set to 4 MPa before the valve and 0.1 MPa after the valve.

**Figure 20:**
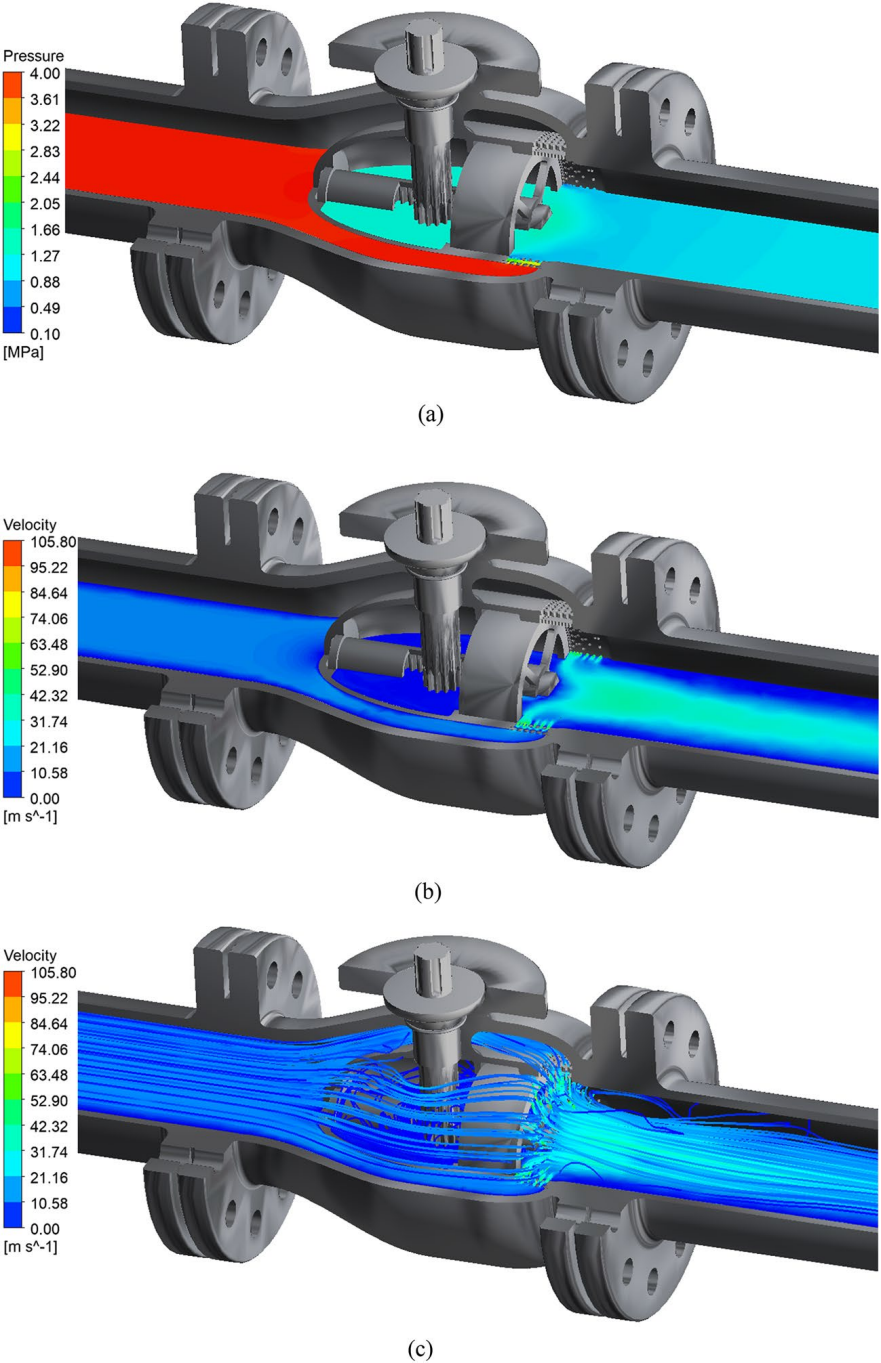
LES simulation results at 100% opening. (**a**) The actual working condition 100 % opening pressure cloud diagram. (**b**) The actual working condition 100 % opening speed cloud diagram. (**c**) The actual working condition 100 % opening speed streamline diagram.

LES is used to simulate the internal flow of the axial flow control valve under actual working conditions. The simulation results on the horizontal section of the 100% opening internal are shown in Fig. 20. From the pressure distribution cloud map of Fig. 20a, it can be seen that the fluid pressure decreases rapidly from 4 MPa to about 0.8 MPa after the fluid passes through the multi-stage pressure-reducing sleeve of the valve. From the velocity streamline distribution cloud map of Fig. 20c, it can be seen that the fluid converges at the middle cavity behind the valve, and the peak velocity of the fluid in the multi-stage pressure-reducing sleeve reaches 103.45 m/s. After the fluid flows out of the pressure-reducing sleeve, the analysis of the streamlined trajectory shows that the fluid intersects and collides, which leads to the rapid decrease of the pressure and velocity of the fluid medium, and then flows behind the valve.

**Figure 21:**
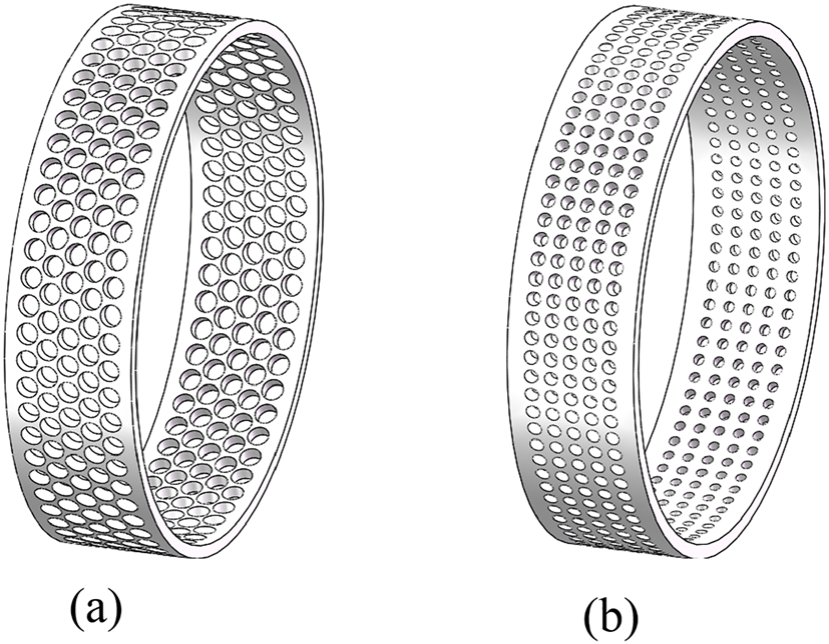
Comparison of the outer sleeve before and after structure optimization. (**a**) Outer sleeve before structure optimization. (**b**) Outer sleeve after structure optimization.

A comparison of the outer sleeve structure of the multi-stage buckling sleeve before and after optimization is shown in Fig. 21.

**Figure 22:**
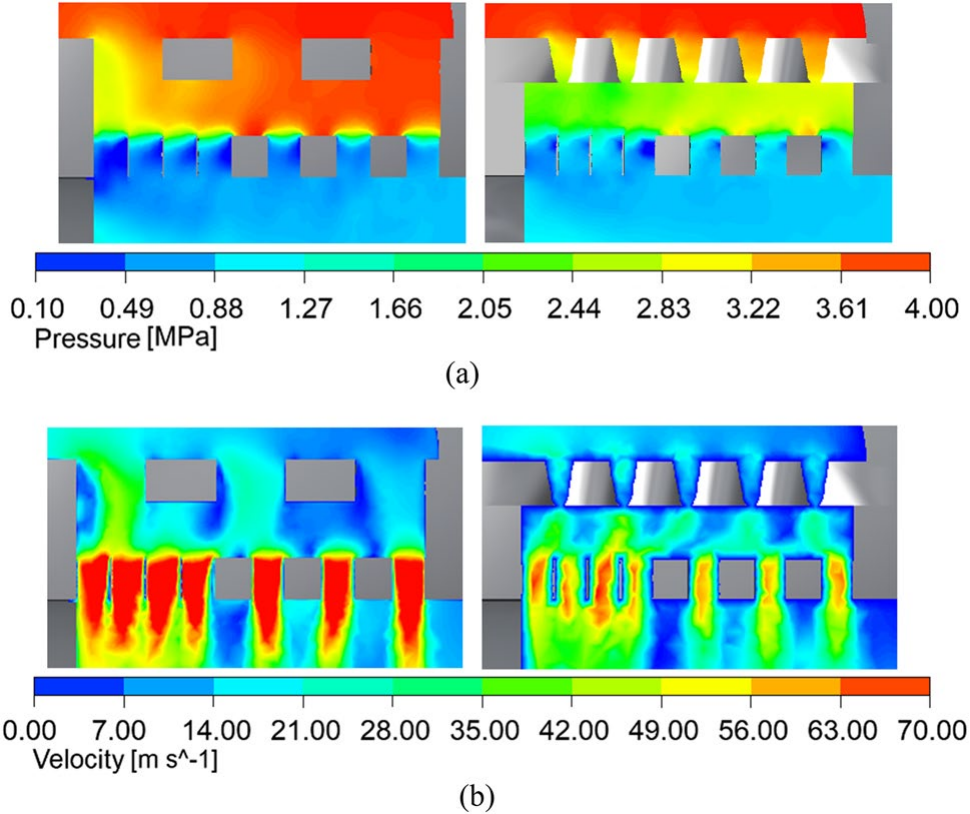
Comparison of local zoomed-in clouds before and after structure optimization. (**a**) Pressure cloud comparison. (**b**) Speed cloud comparison.

As can be seen from Fig. 21, the number of openings in the outer sleeve after structural optimization is increased compared with that before optimization, the diameter of the holes has been reduced, and the thickness of the outer sleeve has increased slightly. The local amplification of the structure before and after optimization is shown in Fig. 22.

From Fig. 22a, it can be seen that the pressure change of the axial flow control valve after the optimization structure is more uniform than that before the optimization. The fluid pressure after passing through the outer sleeve is greatly reduced, but there is still a large amount of high-pressure fluid trapped between the two sleeves before optimization. From Fig. 22b, it can be seen that the speed change of the axial flow control valve after the optimization structure is also greatly improved. Before the optimization, most of the high-speed fluid still flowed into the valve, which may be the main cause of the noise. In the optimized structure, the speed of high-speed fluid decreases rapidly after leaving the influence area of the sleeve throttling effect.

**Figure 23:**
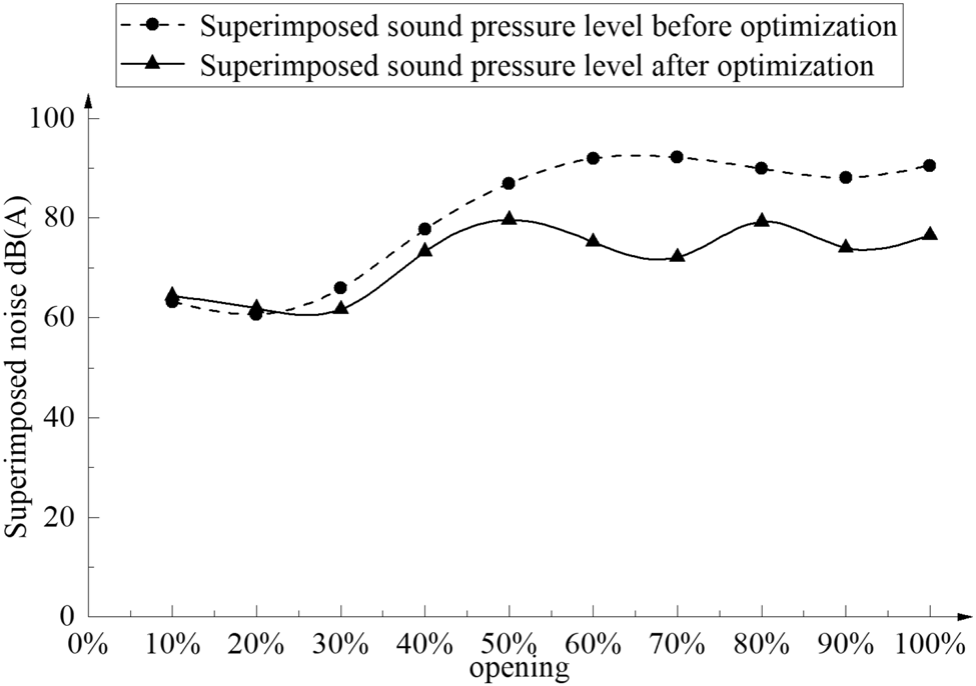
Comparison of superimposed sound pressure levels before and after optimization.

The liquid dynamic noise prediction method of the axial flow control valve is used to predict the noise of the optimized axial flow control valve. The noise values at different frequencies received at the noise receiving point are calculated by the weighted noise calculation formula. The superimposed sound pressure level at the receiving point is calculated, and the superimposed sound pressure level at the receiving point under different openings after optimization is obtained, which is compared with the superimposed sound pressure level at different openings before optimization, as shown in Fig. 23.

It can be seen from Fig. 23 that under a large opening, the noise superposition sound pressure level after structural optimization decreases, and the peak value is 78.2 dB(A), which is significantly lower than that before optimization, and the opening is about 78%. Then the LES large eddy simulation is used to simulate the internal flow field of the axial flow control valve at 78% opening, and the liquid dynamic noise is calculated. The result is 77.8 dB(A), indicating that the optimization method can be used to reduce the noise superposition sound pressure level of the axial flow control valve.

**Figure 24:**
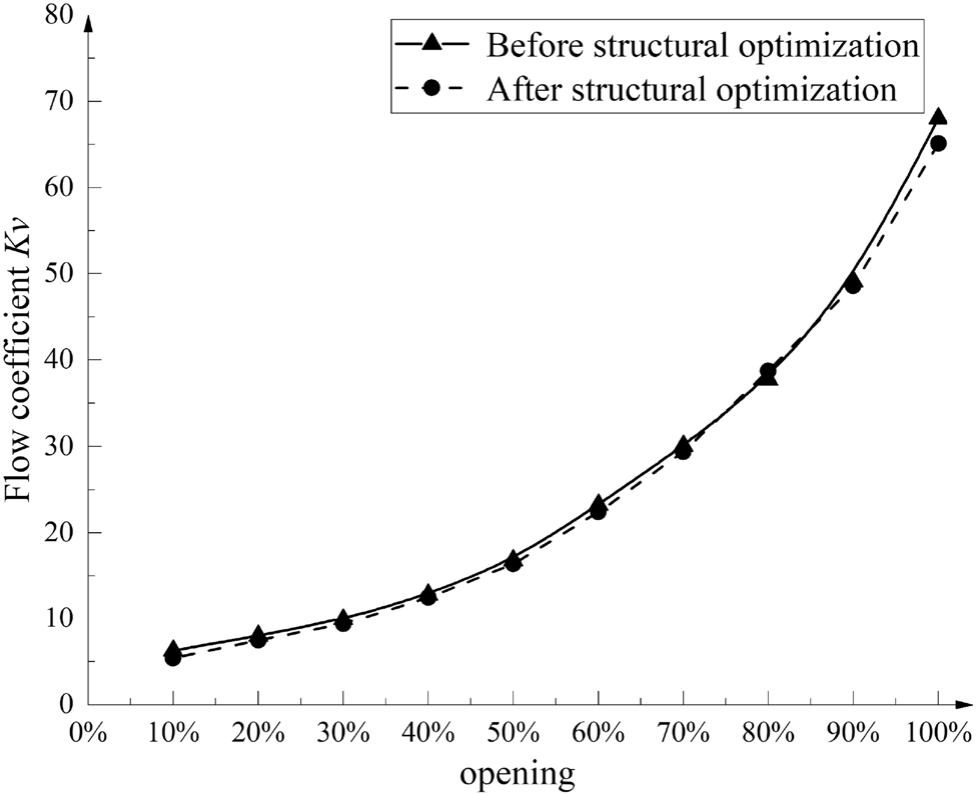
Flow characteristic curve of the axial flow control valve after structure optimization.

The flow value of the axial flow control valve under the typical opening of the test condition after the structural parameters of the multi-stage step-down component are optimized is simulated, and the flow characteristic curve is fitted after the *K*_*v*_ value is calculated. The fitting results are shown in Fig. 24.

From Fig. 24, it can be seen that the flow characteristic curve of the axial flow control valve after structural optimization still maintains the percentage flow characteristic, and the flow capacity is lower than that before optimization, but it still meets the use requirements.

## Conclusion

Based on LES for flow field calculation and noise prediction of axial flow control valves, structural optimization based on Stacking integrated learning and PSO optimization seeking method is proposed to improve the performance of axial flow control valve. The specific conclusions can be summarized as follows:

1. The Stacking algorithm is used to fuse the three machine learning algorithms of decision tree regression, Kriging surrogate model, and support vector regression to learn from each other and build a fusion model with higher accuracy. The accuracy of the fusion model is evaluated by three different error metrics: *R*^2^, RMSE, and MAE. The flow value *R*^2^ is 0.9734, the noise value *R*^2^ is 0.9876, and the reliability is close to 1, indicating that the established Stacking fusion proxy model can well predict the performance of the axial flow control valve.
2. Based on the PSO particle swarm optimization algorithm, the Stacking fusion model is optimized to obtain the best combination of structural parameters that meet the design requirements. The optimized multi-stage depressurization structure parameters are as follows: hole diameter *t*_1_ = 3.8 mm, hole spacing *t*_2_ = 1 mm, hole drawing angle *t*_3_ = 6.4°, hole depth *t*_4_ = 3.4 mm, and two-layer throttling sleeve spacing *t*_5_ = 4 mm.
3. The pressure change of the axial flow control valve after the optimization structure is more uniform than that before the optimization. The fluid pressure after the outer sleeve is greatly reduced, and the speed change is also greatly improved. The speed of the high-speed fluid decreases rapidly after leaving the influence area of the sleeve throttling effect.
4. The peak noise of the axial flow control valve before the optimization of the multi-stage step-down structure is 91.32 dB(A), and it is 78.2 dB(A) after optimization, which is about 14.4% lower than that before optimization. After optimization, the noise level of the axial flow control valve meets the design requirements under most opening degrees. The flow characteristic curve of the axial flow control valve after structural optimization still maintains the percentage flow characteristic and meets the requirement of flow capacity *K*_*v*_ ≥ 60 at the maximum opening.

Compared with the single surrogate model or artificial optimization design, this method has good applicability in the structural optimization of axial flow control valves. It provides a new idea for improving the performance of axial flow control valves in the industry.

Some limitations should be further studied in the following work:

1. In the process of structural optimization of the axial flow control valve, a mature particle swarm global optimization algorithm is adopted. In the follow-up work, the algorithm will be improved according to the characteristics of the axial flow control valve, so that it has more accurate optimization results and faster optimization efficiency.
2. Limited by the test conditions, although the CFD simulation has high accuracy, due to the lack of experimental verification, it may weaken the credibility of the axial flow control valve structure optimization analysis. At present, the product has reached a cooperation intention with related enterprises, and the optimization results will be verified by experiments in the follow-up work.

## Data Availability

The datasets generated during and/or analyzed during the current study are available from the corresponding author upon reasonable request.
